# Antibiotic-resistant bacteria in bovine milk in India

**DOI:** 10.5455/javar.2023.j648

**Published:** 2023-03-31

**Authors:** Sonali Sahoo, Manas Ranjan Behera, Biswajit Mishra, Priyadarshini Sahoo, Sonali Kar

**Affiliations:** 1 School of Public Health, Kalinga Institute of Industrial Technology (KIIT) Deemed to be University, Bhubaneswar, India; 2 Quality Assurance, Kalinga Institute of Industrial Technology (KIIT) Deemed to be University, Bhubaneswar, India; 3 Veterinary Epidemiology and Preventive Medicine, Odisha University of Agriculture and Technology, Bhubaneswar, India; 4 Department of Community Medicine, Kalinga Institute of Medical Sciences (KIMS), Kalinga Institute of Industrial Technology (KIIT) Deemed to be University, Bhubaneswar, India

**Keywords:** Antibiotic residues, mastitis, mitigation strategies, resistant strains

## Abstract

Antibiotic resistance (ABR) is a global issue that draws the attention of all healthcare experts in the veterinary and medical fields. Of various factors, indiscriminate and unregulated antibiotic usage in the animals reared for food production, especially in cows and buffaloes suffering from mastitis, contribute significantly to the rising incidence of resistant bacteria. A literature survey reveals the spread of resistant strains of mastitis-causing bacteria, like *Staphylococcus aureus* and *Escherichia coli*, to humans. In addition, antibiotic residues detected in milk samples against all major groups of antibiotics are likely to enter the human body through the food chain and aggravate the condition. The cumulative effects of ABR have emerged as a silent killer. The benefits of systematic surveillance on ABR in India are yet to be available. Here is an attempt to understand the ABR burden in India associated with bovine milk and its mitigation strategies.

## Introduction

Antibiotic resistance (ABR) is a complex progressive health problem that involves man, animals, and the environment. It has become a serious health issue in the 21st century, threatening life on Earth. The phenomenon occurs when pathogenic microbes are non-responsive to the killing or inhibitory property of standard doses of antimicrobials [1]. Numerous ecological and evolutionary factors contribute to drug-resistant infections [2]. ABR becomes more detrimental when associated with food-producing animals [3]. Large ruminants like cattle and buffalo take a substantial share of ABR for being the reservoir of resistant strains [4]. Bacteria commonly associated with disease called mastitis, such as *Staphylococcus aureus*, *Escherichia coli*, *Pseudomonas* spp., *Proteus* spp., *Klebsiella* spp. etc., and antibiotic residues in milk following therapy [5,6] can be attributed as the major causes. 

Milk is a staple food in Indian households. Dairy farmers are pressured to boost milk production to meet its rising demand, driven by high population growth, rising income, and urbanization [7]. There exists a positive correlation between high milk yield and mastitis [8]. Mastitis is predominantly a bacterial disease of the mammary gland of high-yielders [9]. Antibiotics are often used or misused in the dairy sector for therapeutic and prophylactic purposes [10]. Antibiotics are also eliminated in the milk till 7 days post-treatment [11]. However, there is an absence of compliance to milk withdrawal periods following antibiotic administration, which leads to antibiotic residues in milk [12]. Hence, improper use of antibiotic therapy in milch animals poses a threat to the public, especially if milk is consumed unpasteurized [13].

The consequences of ABR are speculated to be more severe in low-and middle-income countries like India, where the burden of infectious diseases and the use of antimicrobials thereof is high [14]. The issue is further complicated by poverty, illiteracy, overpopulation, and starvation, all of which have contributed to developing drug resistance. Based on these facts, India is often considered the “antimicrobial resistance capital of the world” [15]. The spike in mortality and higher treatment expenses are visible effects of infection with resistant bacteria [16,17]. Numerous diagnostic and therapeutic challenges arise with developing newer multi-drug resistant (MDR) strains [18,19]. 

The period from 2000 to 2010 saw a 76% increase in global antibiotic consumption by Brazil, Russia, India, China and South Africa [20], of which a 23% increase was attributable to India alone. Although the dairy sector is often considered one of the major drivers of agriculture-related ABR, the linkage between antibiotic use in the veterinary sector and ABR pathogens is not fully established [21]. 

Available literature gives a clear message on the magnitude of ABR in the global sphere [4,22,23]. However, the data from such studies cannot be extrapolated to give a clear picture of ABR in India, a prerequisite to preparing a strong strategy to combat this life-threatening health issue. To use antibiotics appropriately and maintain the therapeutic arsenal, which ensures the medicines’ long-term efficiency, it is important to estimate the level and trend of resistance in udder infections. In this context, a review was undertaken to assess the status of resistant bacteria and antibiotic residues detected in the milk of dairy cows across India. We also highlight the impact of such resistant strains on public health and mitigation approaches.

## Possible Routes of Entry of Pathogens into Milk 

**Figure 1.**
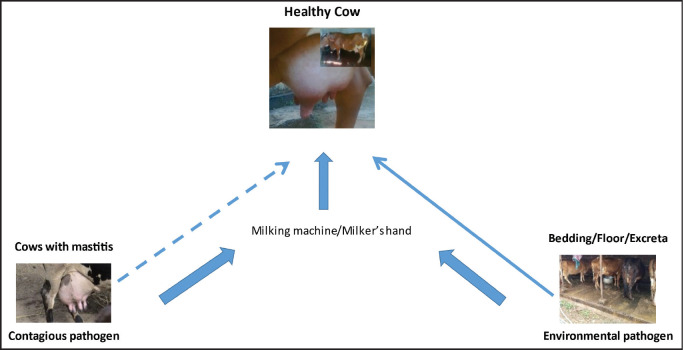
Possible routes of entry of bacteria to udder of bovines.

Milk, a sterile component in healthy udder cells, is later colonized by pathogenic bacteria from various sources (Fig. 1). The high nutritional value of bovine milk, combined with its near-neutral pH, creates a favorable medium for developing numerous microorganisms [24]. The pathogens causing udder infections, popularly known as mastitis, can be categorized as contagious and environmental [25]. Bacteria in the mammary glands of infected cows are classified as a contagious category, viz., *S. aureus*, *Streptococcus agalactiae*, *Corynebacterium bovis*, and *Mycoplasma bovis* [26,27]. These bacteria may enter the teat orifice of a healthy cow through the milker’s hand or equipment contaminated with these bacteria [8,28]. Another group of bacteria classified as environmental such as coliforms, *Klebsiella* spp., *Streptococcus dysgalactiae*, *Streptococcus uberis*, etc., are present in the cow’s environment, which includes bedding, floor, dung, etc. [9] and may directly enter the teat. The teat sphincter remains dilated for about 1–2 h post-milking, facilitating the entry of pathogens [29]. High stocking density combined with unhygienic management practices, the large population of multiparous older cows, pendulous udders, lack of udder and leg hygiene, teat end morphology, and milking mastitis-affected cows before healthy cows are the key risk factors linked to the development of intramammary infections [30,31]. Moreover, it is crucial to monitor cows’ health during the dry period of the lactation cycle because any new intramammary infection will influence the subsequent lactation [8]. 

## ABR Pathogens in Milk Samples Across India 

The pathways that lead to resistance are enzymatic degradation, target site alteration, modification of the permeability of the bacterial cell wall, and alternate paths to escape the activity [32]. The resistance determinants are transferred through one of the pathways, such as (1) modification of the existing genes (vertical gene transfer) and/or (2) acquisition of genes from bacteria present in the environment (horizontal gene transfer) [4]. 

Antimicrobial resistance genes (ARGs) are thought to cause phenotypically expressed ABR. Some key genes leading to ABR include *blaCTX-M*, *blaSHV*, *blaTEM*, *mec*(A), and *blaZ* genes for the β-lactam group of antibiotics [33,34]; *van* genes for glycopeptides [35]; *gyrA*, *gyrB*, *parC,* and *parE* genes for quinolones [34]; *folP*, *sul1*, *sul2,* and *sul3* genes for sulphonamides [36]; *tet* genes for tetracyclines [35] and erm gene for macrolides [37]. ARGs are detected through phenotypic and genotypic methods [38,39] and metagenomic deep sequencing [40]. Metagenome study is the cutting-edge technology used across the world for the detection of resistant genes in milk [41–44]. In a similar study on Kankrej, Gir, and crossbred cows of Gujarat state, genes resistant to fluoroquinolones and methicillin were detected in milk [45]. 

Antibiogram studies conducted in bovine milk have shown variable results against pathogens inhabiting the udder [46–50]. According to a recently published meta-analysis report, *Staphylococcus* spp. emerged as the most prevalent mastitis-causing bacteria, causing 45% of cases in India [51]. *In vitro* studies revealed that penicillin, streptomycin, erythromycin, tetracycline, ampicillin, and cephalothin developed resistance against *S. aureus* in cows suffering from mastitis [52]. Pure isolates of *S. aureus* from milk samples of lactating cows exhibited resistance against norfloxacin, penicillin, ciprofloxacin, vancomycin, nalidixic acid, and ampicillin [53]. In a similar study, *S. aureus* isolates showed resistance to various β-lactams like penicillin, amoxicillin, and methicillin, as well as oxytetracycline [54]. In a study, it was observed that some isolates of *S. aureus* from mastitis milk samples of cows were resistant to all antibiotics tested viz, ampicillin, amoxicillin-sulbactam, ceftriaxone, enrofloxacin, methicillin, and penicillin [55]. Additionally, resistance profiling against various antibiotics revealed that most *S. aureus* isolates were resistant to multiple antibiotics [56]. Given that the *S. aureus* isolates were resistant to more than three antibiotics, they were MDR bacteria. More than 50% resistance to beta-lactam, macrolide, sulfa drugs, and the tetracycline group of antibiotics was seen in bacterial isolates [57]. In a similar study, the resistance pattern of *S. aureus* to ampicillin, methicillin, oxacillin, ceftriaxone, rifampicin, vancomycin, amoxiclav, oxytetracycline, erythromycin, nitrofurantoin, cefuroxime, gentamicin, norfloxacin, ciprofloxacin, and levofloxacin was observed in milk of cows and buffaloes [58]. Milk samples of cows suffering from Staphylococcal mastitis showed resistance against gentamicin, clindamycin, erythromycin, rifampicin, tetracycline, oxacillin, cefoxitin, and teicoplanin [56]. The above resistance patterns indicate the resistance of *S. aureus* to several antibiotics over the years. Additionally, *E. coli* (14%) and *Streptococcus* spp. (13%) also contribute significantly [51]. 

The *mecA* gene that encodes the penicillin-binding protein 2a and has a lower affinity for penicillinase-resistant penicillin mediates methicillin resistance in *Staphylococcus* spp. [59]. Methicillin-resistant *S. aureus* (MRSA) in the milk of bovines showed resistance to methicillin, amoxicillin, penicillin, and oxytetracycline [54]. As per an *in vitro *antibiotic sensitivity testing, MRSA was completely resistant to methicillin and other members of the penicillin group of antibiotics [60]. Due to the availability of other similar antimicrobials, MDR bacteria tend to be resistant to certain antibiotics even though the resistant antibiotics are not present in that environment [61]. Widespread intramammary broad-spectrum antibiotic therapy and the production of beta-lactamase, an enzyme that renders penicillin and closely related antibiotics inactive, may be responsible for the resistance of *S. aureus* in intramammary infections [62]. The ability of *S. aureus* to form biofilms delays the penetration of antibiotics [63]. Increased resistance to penicillin and other semi-synthetic antibiotics such as macrolides, tetracyclines, and aminoglycosides have made treating Staphylococcal illness a global problem [64].

Drug resistance has also been detected against *E. coli *isolates to penicillin, amoxicillin, oxytetracycline, and methicillin [54]. The phenotypic antibiotic resistance pattern of *E. coli *isolates from bovine milk samples revealed resistance to levofloxacin, penicillin-G, cefoxitin, cefotaxime, ampicillin, ceftazidime, ceftizoxime, co-trimoxazole, and ceftazidime [65]. Shiga-toxigenic *E. coli* (STEC), found in raw milk samples, was fully resistant against penicillin, cefalexin, rifampicin, methicillin, and novobiocin. This study also highlights the risk of virulent MDR STEC in raw milk, which stresses the need for routine surveillance programs [66]. 110 bacterial isolates of 14 different taxa have been observed from bovine mastitis cases. Gram-positive bacteria are resistant to vancomycin and penicillin, and Gram-negative bacteria are resistant to numerous drugs, including extended-spectrum lactamases, cephalosporins, tetracyclines, vancomycin, and chloramphenicol [22]. Vancomycin-resistant enterococci, MDR Gram-negative rods, MDR *Pseudomonas *spp., cephalosporin-resistant *S. uberis,* and MDR *Acinetobacter* spp. were also recorded in the above study.

In a study conducted on bubaline milk samples of Karnataka, variation in resistance pattern observed against *S.* aureus, coagulase-negative Staphylococci (CoNS), and *E. coli *is mentioned subsequently [67]*. S. aurues* isolates were resistant to cefoxitin, penicillin-G, and ceftriaxone/sulbactam. On the contrary, resistance to methicillin, amoxicillin/sulbactam, and penicillin-G were observed in CoNS. Resistance to methicillin, streptomycin, cefoxitin and penicillin-G was observed in the antibiogram of *Streptococci* isolates. Interestingly, maximum resistance was observed against *E. coli* isolates. Drug sensitivity pattern examination of bovine milk revealed resistance to streptomycin, penicillin G, ampicillin, cloxacillin, amoxicillin, and neomycin against a wide range of Gram-positive and Gram-negative organisms [68]. Most isolates in milk samples of crossbred cows have developed resistance to penicillin G, a commonly available and routinely used antibiotic [69,70]. In a similar study conducted in and around Meerut, *Staphylococcus* spp. (42.55%) was the predominant isolate in bovine mastitis milk followed by *E. coli* (21.28%), *Proteus* spp. (8.51%), *Streptococcus* spp. (6.38%) and mixed infection (8.09%). Furthermore, maximum resistance was observed against amoxiclav and ampicillin/cloxacillin in that study [71]. 

Resistance of mastitis-causing pathogens to antibiotics present in the “reserve” category as per the World Health Organization (WHO)-Access Watch Reserve classification indicates a grave threat [72]. This category includes “last resort” antibiotics for treating confirmed or suspected infections caused by human MDR pathogens. Such transfer of MDR pathogens could threaten public health and the dairy industry. Table 1 shows some important resistance genes identified from bovine milk samples in India.

## Antibiotic Residues in Bovine Milk

Mastitis plays a major role in antibiotic residues in milk and the subsequent development of ABR. Milk often contains residues of antibiotics following its administration through intramammary and parenteral routes [79]. As per the European Commission, antibiotic residues are “pharmacologically active substances and their metabolites which remain in foodstuffs obtained from animals to which the veterinary medicinal products have been administered.” There is a milk withdrawal period that extends to 7 days following antibiotic therapy [11]. However, it is rarely practiced by farmers due to ignorance about its adverse health impacts and/or financial losses associated with milk discard [80]. Table 2 depicts the scenario in India concerning the antibiotic residues detected in bovine milk samples. 

Different antibiotics have maximum residual concentrations defined such that no unforeseen negative effects will likely result from these medications. Considerable progress has been made in detecting antibiotic residues by chromatographic, immunological, and microbiological techniques [79]. The best ways to avoid consuming this contaminated milk are to discard the milk until the recommended withdrawal period and to minimize the use of antibiotics. The primary reason for its presence in milk is incorrect antibiotic use for treating diseases, especially mastitis [92]. 

## Impact of ABR on Public Health

The consumer is directly at stake when resistant pathogenic microorganisms and/or antibiotics are present in the food chain. As per the WHO data, 700,000 deaths are caused by MDR bacteria across the globe annually. Among different age groups, the geriatric population commonly suffers from infections of the respiratory system, urinary tract, soft tissues, and skin and becomes chronically ill and immunocompromised [93]. MRSA, vancomycin-resistant *S. aureus*, extended-spectrum beta-lactamase-producing Enterobacteriaceae, carbapenem-resistant Enterobacteriaceae, and resistance to colistin threaten the lives of newborns [94]. Immunocompromised patients (suffering from human immunodeficiency virus and acquired autoimmune deficiency syndrome) and organ transplant patients suffer from diseases caused by MDR pathogens [95]. 

*Staphylococcus *spp. remains one of the leading causes of food-borne illnesses [96]. Livestock-associated MRSA has been reported to cause nasal, skin, and soft tissue infections in humans. Furthermore, heat-resistant enterotoxins are produced by *S. aureus* and causes gastrointestinal discomfort when consuming infected milk [97]. *Streptococcus agalactiae*, an important mastitis pathogen, causes bacteremia, skin infection, soft tissues, and urinary tract with necrotizing inflammation of various internal organs [98]. Investigations revealed that young dairy cattle are reservoirs of enterohemorrhagic *E. coli* (EHEC) O157:H7, a new source of foodborne disease, and hemorrhagic colitis (bloody diarrhea) and hemolytic uremic syndrome are two conditions caused by infection with EHEC strains [99]. It raises concern as these pathogens enter the food chain and pose a threat to consumers [74].

**Table 1.** Resistance genes identified from bovine milk samples in India.

Gene(s)	Organism(s)	State(s)
*mecA *and* blaZ*	MRSA	Tamil Nadu [73]
*blaCTX-M, blaTEM, blaSHV, qnrS, qnrB *and* sul1*	*K. pneumoniae*	West Bengal, Jharkhand and Mizoram [74]
*mecA *and* blaZ*	*S. aureus* and coagulase negative Staphylococci	Kerala [75]
*blaTEM, blaSHV, blaCTX-M, tet(A),tet(B) *and* tet(C)*	*E. coli*, *Pseudomonas* spp., *Proteus* spp., *Klebsiella* spp. and E*nterobacter* spp.	West Bengal [76]
*blaCTX-M*	*E. coli*	West Bengal [77]
*blaAmpC, sul1, sul2 *and* qnrS*	*E. coli*	West Bengal [65]
*bla CTX-M, bla SHV *and* bla TEM*	*E. coli*	Gujarat [78]
*mecA*	MRSA	Telangana, Andhra Pradesh and Tamil Nadu [56]

**Table 2.** Antibiotic residues detected in bovine milk in India.

State/region	Antibiotics detected in milk
Northwestern Himalayan region [81]	Oxytetracycline and amoxicillin
Gujarat [82]	Tetracycline, fluoroquinolones, gentamicin
Kerala [83]	Oxytetracycline
Karnataka [84]	Tetracycline and azithromycin
Punjab [85]	Tetracycline, enrofloxacin, and oxytetracycline
Punjab [86]	Oxytetracycline, enrofloxacin and penicillin G
Kerala [87]	Tetracycline, ß-lactams and enrofloxacin
Kerala [88]	Tetracycline and ß-lactams
Bihar [89]	Tetracycline, oxytetracycline, sulfadimidine and sulfamethoxazole
Andhra Pradesh [90]	Tetracycline and ß-lactams
Punjab [91]	Tetracycline

**Table 3.** Proposed mitigation approaches to be addressed by different stakeholders.

Stake holders	Proposed actions
Dairy farmers	Preventive/prophylactic measures against infectious diseases Adherence to the antibiotic withdrawal period
Veterinary and medical fraternity	Non-antibiotic approaches as an adjunct to antimicrobial therapy Judicious use of drugs based on antibiotic susceptibility test (ABST) Regular monitoring of ABST results and their sharing
Pharmaceuticals	Avoid selling or dispensing of antibiotics over the counter without a prescription Sharing of data on antimicrobial production and sales with the government
Scientists	Conduct routine surveillance studies on antimicrobial use and AMR Analysis of the current trends in resistance to guide the government in framing evidence-based policies Strengthening research on economical and reliable alternative strategies to combat AMR (probiotics, prebiotics, synbiotics, phytocompounds,phage therapy, nanoparticles, bacteriocins, peptides, immunostimulants, cytokines, Quorum Quenchers, enzyme therapy, CRISPR-Cas) Develop novel point-of-care diagnostics for quicker detection of resistance
Government	Strict vigilance on the over-the-counter sale of antibiotics Regular interaction of all stakeholders on AMR to review the status of various intervention strategies and decide the future course of action
Consumers/Public	Awareness among the public/consumer on the adverse impact of AMR though television, newspapers and social media

The presence of antibiotic residues has both short and long-term impacts on the health of individuals [100]. Hypersensitivity is the major short-term impact observed, especially with beta-lactams, whereas long-term exposure will lead to carcinogenicity, mutagenicity, teratogenicity, and disruption of gastrointestinal microflora [101]. Also, there is a concern that antibiotic residues in milk may drastically alter the microbial population of the human gastrointestinal system [102].

## Mitigation Strategies to Combat ABR

As per the Economic survey 2021–2022, India is the world’s top milk producer, with a compound annual growth rate of about 6.2%. Numerous efforts have been made in India to address the issue of antibiotic resistance, including national policy for antimicrobial resistance (AMR) containment, the “Chennai Declaration,” the “Jaipur Declaration on Antimicrobial Resistance,” the National Action Plan (NAP) on AMR of 2017, and the “Redline” campaign [80]. In 2017, Food Safety and Standards Authority of India (FSSAI) published the tolerance limits for antibiotics in foods of animal origin [103]. In the same year, surveillance of AMR in food animals and aquaculture was initiated by the Indian Council of Agricultural Research [103]. It determined the pattern of resistance indicators and pathogenic bacteria isolated from food animals. The initiatives taken by the Indian Government, such as the NAP-AMR and FSSAI-set antibiotic residual limits in food from animal origin, attempt to address important antibiotic policy and regulatory challenges in line with the principles of One Health [4,104]. However, the pace of implementation at the community level is considered inadequately regulated and monitored. ABR is inherently a global problem that can only be controlled at a supra-national scale by mitigation approaches encompassing multiple stakeholders (Table 3). Filling information gaps, cautiously piloting initiatives, and meticulously evaluating successes and failures are thus the need of the hour to combat this menace. 

## Conclusion

Antibiotic resistance is a life-threatening global health issue affecting humans and animals, and India is not free from this pandemic. ABR has proved more harmful to humans when it originated in food-producing animals as the residual antibiotic and the resistant bacteria spread quickly to multiple hosts, including humans, through milk. Thus, a robust strategy involving all stakeholders is essential to put break in the development of new MDR strains and in reducing morbidity and mortality associated with ABR. 

## List of abbreviations

ABR, Antibiotic resistance; AMR, Antimicrobial resistance; MDR, Multi-drug resistant; ARGs, Antimicrobial resistance genes; MRSA, Methicillin-resistant *S. aureus*; STEC, Shiga-toxigenic *E. coli*; CoNS, Coagulase-negative Staphylococci; WHO, World Health Organization; EHEC, Enterohemorrhagic *E. coli*; NAP, National Action Plan; FSSAI, Food Safety and Standards Authority of India; ABST, Antibiotic susceptibility test; AMU, Antimicrobial use.
